# Improving Tree-Based Lung Disease Classification from Chest X-Ray Images Using Deep Feature Representations

**DOI:** 10.3390/bioengineering13030267

**Published:** 2026-02-25

**Authors:** Abdulaziz A. Alsulami, Qasem Abu Al-Haija, Rayed Alakhtar, Huda Alsobhi, Rayan A. Alsemmeari, Badraddin Alturki, Ahmad J. Tayeb

**Affiliations:** 1 Department of Information Systems, Faculty of Computing and Information Technology, King Abdulaziz University, Jeddah 21589, Saudi Arabia; aaalsulami10@kau.edu.sa; 2 Department of Cybersecurity, Faculty of Computer & Information Technology, Jordan University of Science and Technology, P.O. Box 3030, Irbid 22110, Jordan; 3 Department of Information Technology, Faculty of Computing and Information Technology, King Abdulaziz University, Jeddah 21589, Saudi Arabia; ralakhtar@kau.edu.sa (R.A.); ralsemmeari@kau.edu.sa (R.A.A.); baalturki@kau.edu.sa (B.A.); ajtayeb@kau.edu.sa (A.J.T.); 4 Department of Information Technology, Faculty of Computing and Information Technology, Taif University, Taif 21974, Saudi Arabia; hasobhi@tu.edu.sa

**Keywords:** tree-based classifiers, fine-tuned CNN, deep feature extraction, chest X-Ray, lung disease screening

## Abstract

Healthcare systems worldwide face increasing pressure to deliver accurate, affordable, and scalable diagnostic services while maintaining long-term sustainability. Chest X-ray screening is considered one of the most cost-effective methods for detecting lung disease. However, many deep learning approaches are computationally intensive and difficult to interpret, which limits their adoption in high-throughput, resource-constrained clinical settings. This study proposes a hybrid CNN–tree framework for automated lung disease classification from chest X-ray images, which targets COVID-19, pneumonia, tuberculosis, lung cancer, and normal cases. To ensure robustness and generalization, four publicly available chest X-ray datasets from different sources are merged into a unified five-class dataset, which introduces realistic variations in imaging conditions and patient populations. A ResNet-18 model is fine-tuned to extract domain-specific deep feature representations. Feature dimensionality and redundancy are reduced using Principal Component Analysis, while class imbalance is addressed through the Synthetic Minority Over-sampling Technique. The resulting compact feature vectors are used to train interpretable tree-based classifiers, which include Decision Tree, Random Forest, and XGBoost. Experiments conducted using five-fold stratified cross-validation demonstrate substantial and consistent performance gains. When trained on fine-tuned and preprocessed deep features, all evaluated tree-based classifiers achieve weighted F1-scores between 0.977 and 0.982 using five-fold cross-validation, with a significant reduction in inter-class confusion. In addition, the proposed framework maintains low per-sample inference latency, which supports energy-efficient and scalable deployment. These results indicate that combining deep feature learning with interpretable tree-based models provides a practical and reliable solution for sustainable chest X-ray screening in real-world clinical environments.

## 1. Introduction

The interpretation of chest X-ray (CXR) images is a foundation of pulmonary medicine, but it remains one of the most challenging tasks in diagnostic radiology [1]. Tuberculosis continues to be a global health priority, which represents a significant cause of mortality and morbidity risk, particularly in regions with limited access to expert radiologists [2,3].

Additionally, the need for rapid multi-class diagnostic tools has increased due to the emergence of COVID-19 and the growing prevalence of chronic diseases, including lung cancer and chronic obstructive pulmonary disease (COPD) [4,5].

Although CXRs are accessible and cost-effective, their interpretation is subjective and prone to inter-observer variability [3,6]. In this context, automated diagnostic systems (ADS) driven by artificial intelligence (AI) become important in providing accurate detection and classification [3,7]. Over the last decade, Deep Learning (DL) models, particularly Convolutional Neural Networks (CNNs), have shown effectiveness in medical image classification [8]. Modern architectures such as ResNet-18 and ResNet-50 can extract complex spatial features indicative of different lung diseases [9,10]. However, another challenge that has emerged as AI moves from research to clinical settings is the black-box nature of deep neural networks [11]. Clinicians often find it challenging to accept or act upon complex AI-generated diagnoses [11,12]. To increase the interpretability and dependability of AI-based decisions in clinical practice and ensure that healthcare providers can understand the logic behind diagnostic results, there is a need for more interpretable AI [12,13].

Recent studies have focused on hybrid frameworks that integrate interpretable tree-based and ensemble classifiers with deep representation learning to address this gap [14]. Such frameworks are essential to the development of sustainable healthcare innovations because they balance the accuracy of diagnosis with the minimal processing power required for implementation in environments with limited resources [15]. Tree-based models such as Random Forest, XGBoost, and the Natural Gradient Boosting provide fundamental interpretability by providing feature importance scores and detailed decision paths [14]. Despite these advantages, raw high-dimensional pixel data usually presents challenges for tree-based models [9]. Rajinikanth et al. [9] proposed a strategy that effectively transforms unstructured picture data into a classification feature set suitable for ensemble learning by using a fine-tuned ResNet-18 model to extract domain-specific features. The effectiveness of any medical ADS depends on data quality and the independence of the classification algorithm itself. Class imbalance can affect model performance and generalization, as medical datasets are susceptible to bias [4]. For example, in a clinical study, normal patient instances often outperform lung cancer or TB instances. Preprocessing methods like the Synthetic Minority Over-sampling Technique (SMOTE) are used to address class imbalance and maintain the learning process [4]. Furthermore, CNN-generated feature vectors often contain noise and redundancy. Principal Component Analysis (PCA) can be integrated to provide an efficient feature set that retains the most relevant diagnostic characteristics, reducing overfitting and improving tree-based classifier performance [16]. The study in [15] confirms that the hybrid approach provides an accurate and interpretable solution for automated lung disease classification. The integration of feature learning with conventional tree-based models is a sustainable hybrid approach that balances prediction accuracy with the computational capabilities required for sustained implementation in digital health environments.

This paper investigates how deep feature representations can be used to adapt tree-based machine learning models for multiclass chest X-ray analysis while maintaining computational efficiency. The study uses a hybrid learning method in which classical tree-based classifiers make the final decision and convolutional neural networks are only used for feature extraction, rather than end-to-end deep neural networks. Four different public chest X-ray datasets containing COVID-19, pneumonia, tuberculosis, lung cancer, and normal cases are combined into a single multiclass benchmark to ensure robustness and practical use. The proposed method systematically examines the impact of feature quality, preprocessing, and classifier selection on achieving reliable and stable automated lung disease detection through fine-tuning, feature preprocessing, and robust cross-validation. The main contributions of this research can be summarized as follows:

- We propose a hybrid CNN–tree framework for multiclass lung disease classification from chest X-ray images, which combines deep feature learning with classical tree-based models.
- We demonstrate that selective fine-tuning of a ResNet-18 backbone, combined with feature normalization, PCA dimensionality reduction, and class balancing using SMOTE, improves the performance of tree-based classifiers.
- We provide a comprehensive evaluation of Decision Tree, Random Forest, and XGBoost classifiers on four publicly available chest X-ray datasets merged into a unified dataset under consistent experimental conditions. We evaluate the classifiers using stratified five-fold cross-validation and highlight the impact of feature quality on classifier performance.
- We analyze computational efficiency at the per-sample level and show that the proposed framework achieves near-optimal accuracy and F1-score with low inference latency, which supports deployment in real-time and resource-constrained clinical environments.

## 2. Related Work

The automated analysis of chest X-ray images for respiratory disease detection has developed significantly with the introduction of deep learning (DL). The majority of research focuses on Convolutional Neural Networks (CNNs) and Vision Transformers (ViTs) to differentiate between illnesses such as COVID-19, various forms of pneumonia, tuberculosis (TB), and cancer. Several studies have utilized transfer learning with pre-trained architectures to address the limits of restricted medical datasets. For example, modified ResNet-50 and VGG architectures have been successfully used to classify COVID-19 and pneumonia. Hybrid methods, such as combining Inception V3 and VGG16, have been proposed to improve predictive performance while reducing overfitting.

Lu et al. [15] introduces the CTBViT model for the detection of TB in CT and X-ray images. The model addresses inefficiencies in traditional transformer blocks, which consider all tokens for self-attention, by implementing a Patch Reduction Block (PRB) that eliminates less important tokens and focuses on key areas for classification. In addition, a randomized classifier is proposed in which the input layer parameters are randomly initialized and remain unchanged during training, thereby mitigating overfitting. The CTBViT model was validated through experiments on public datasets, namely COVID-19, Pneumonia, and COVID-19 Radiography. They achieved accuracy rates of 98.4% for COVID-19 and Pneumonia and 98.5% for COVID-19 Radiography. Shati et al. [17] developed ETDHDNet that integrates texture analysis with deep feature learning to enhance TB prediction in chest X-rays. The architecture includes an Extended Texture Descriptor Histogram (ETDH) module that captures multi-scale texture details using linear binary patterns. These are fused with hierarchical features from a DenseNet121 network and classified via a multilayer perceptron. The model achieved accuracies of 99.05% on the TBX11K dataset, 99.29% on the Tuberculosis Chest X-ray Database, and 88.05% on the Shenzhen Dataset. A Hasib et al. [16] proposed a framework that combines synthetic image augmentation with DL models to address dataset imbalance and improve diagnostic accuracy using Explainable AI techniques. Their model benchmarks InceptionV3, DenseNet, and ResNet. Synthetic images are generated utilizing Feature Interpolation through Linear Mapping and PCA to enhance the diversity of the dataset called the Covid-19 & Pneumonia dataset with the YOLOv8 and InceptionV3 models fine-tuned in this augmented dataset called Chest X-ray Covid-19 and Pneumonia-Aug-FILM-PCA. Grad-CAM is employed for model explainability, supported by large language models for visualization analysis. The InceptionV3 model achieved an accuracy of 96%, and the YOLOv8 model achieved 97%.

Randieri et al. [18] presented a lightweight CNN-based framework for three-class classification of COVID-19, pneumonia and normal subjects. The framework uses Contrast-Limited Adaptive Histogram Equalization for contrast enhancement, followed by data augmentation to prevent overfitting. Their 19-layer architecture trains around 13 million parameters and uses LeakyReLU to maintain gradient flow. They have evaluated their framework using two public datasets, namely the COVID-19, Pneumonia, and Normal Chest X-ray PA and COVID-19 Radiography Database. The model achieved 97.48% accuracy and demonstrated superior computational efficiency, with an average inference time of $9.92\times 10^{-4}$ s, making it suitable for implementation on embedded hardware platforms such as FPGAs. Alotaibi et al. [19] developed an automated system that uses a modified ResNet-50 pre-trained model to classify CXRs into three categories: COVID-19, normal, and pneumonia, including datasets from King Abdul Aziz Medical City, the covid19-radiography-database, and Chest X-Ray Images, to form a more robust training dataset. The study integrated several datasets, including a local dataset from King Abdul Aziz Medical City, covid19-radiography-database, and Chest X-Ray Images, to form a more robust training dataset. They used split data and 5-fold cross-validation, achieving an accuracy of 95.28%. Wajgi et al. [20] proposed an optimized TB classification system that fuses hyperparameter tuning with transfer learning. They identified optimal configurations using a VGG19 base model with the tanh activation function, a stochastic gradient descent optimizer, and a 0.3 dropout rate. The framework achieved an accuracy of 98.11% in the Tuberculosis Chest X-ray Database. Mirugwe et al. [21] conducted a comparative study of six CNN architectures, including VGG16, VGG19 and various ResNet to improve TB detection. They evaluated their study using a publicly available dataset called Tuberculosis Chest X-ray Database. The study found that VGG16 outperformed other models, achieving an accuracy of 99.4%. Their findings suggest that simpler models can provide an ideal balance between diagnostic accuracy and computational efficiency. The more complex architectures, like ResNet152, required longer training times, like 356.6 min, without proportional performance gains.

Rahman et al. [14] introduced TB-CXRNet, a framework for tuberculosis (TB) detection and drug-resistant TB stratification, trained on the QU-MLG-TB dataset. This dataset was constructed by integrating several public sources, including the Tuberculosis (TB) Chest X-ray Database, RSNA CXR Dataset, PadChest Dataset, NLM Dataset, Belarus Dataset, and the NIAID TB Dataset, resulting in a total of 40,000 CXR images. The model employs a CheXNet-based CNN encoder with a Self-MLP classifier that learns an optimal set of operators during training. For stratification, a stacking-based machine learning model was used to distinguish drug-sensitive TB from drug-resistant TB, including MDR and XDR cases. The proposed framework achieved classification accuracies of 93.32% for TB, 87.48% for binary drug-resistant TB, and 79.59% for three-class drug-resistant TB. Srinivas et al. [22] proposed a hybrid IV3-VGG model that combines Inception V3 and VGG16 for COVID-19 prediction. The architecture uses the first six layers of VGG16 for initial feature extraction, followed by Inception V3 blocks and reduction blocks to avoid representational bottlenecks. The model achieved an accuracy of 98% on the COVID-19 Radiography Database, outperforming DenseNet121 (88%) and MobileNet (91%). El Houby [23] developed a framework for COVID-19 detection using VGG19 and EfficientNetB0 based on transfer learning. The study explored both full-chest X-ray images and lung-segmented images generated via mask multiplication. Results indicated that models trained on full images performed better than those trained on segmented images, due to the presence of relevant diagnostic information in the surrounding lung tissue. They evaluated their model using a publicly available dataset called the COVID-19 Radiography Database. The VGG19 model, when contrast-limited adaptive histogram equalization (CLAHE) was applied to the full images, achieved the highest binary classification accuracy of 95%. Table 1 provides a summary of related works.

Dataset restrictions limit much previous research, as their models were focused on particular disease classifications, such as COVID-19 vs. normal, thereby reducing generalizability to broader clinical settings. Furthermore, the remarkable accuracy claimed by several methods often comes at the cost of interpretability, leading to black-box simulations that undermine clinical transparency. Furthermore, using learning models directly on high-dimensional chest X-ray data often leads to feature redundancy and saturation, reducing learning effectiveness and overall model robustness. Most existing papers focus on individual training datasets rather than integrated, multiclass datasets and place less emphasis on computational cost. In contrast, this work employs multiple datasets in a unified multiclass setting, converts images into vector representations to reduce computational cost, trains on merged datasets to improve generalization, and utilizes a lightweight model-tree-based classifier for efficient, robust performance.

## 3. Methodology

This section describes the proposed methodology for improving the performance of tree-based classification models on chest X-ray images. The proposed framework integrates a CNN for feature extraction with classical tree-based classifiers for the final classification stage. The following subsections detail the system architecture, deep feature extraction process, feature preprocessing strategy, and the tree-based classification models employed in this study.

### 3.1. System Architecture

**Figure 1:**
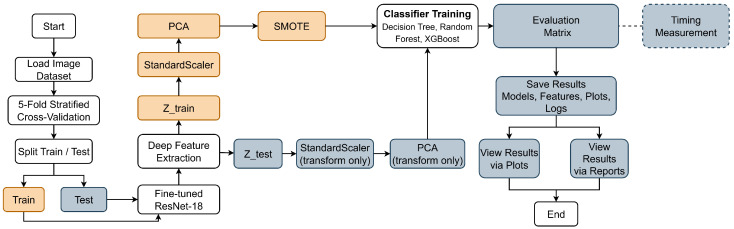
Overall architecture of the proposed deep feature–based framework for improving tree-based classification on chest X-ray images.

The architecture of the proposed framework illustrates the complete processing pipeline of the method. It shows the integration of deep feature extraction with tree-based classifiers for chest X-ray image classification, as shown in Figure 1. The framework is designed as a modular pipeline that sequentially processes input images through feature extraction, preprocessing, classification, and evaluation stages while ensuring reproducibility across experiments.

The process begins by loading the chest X-ray image dataset, which is then partitioned using five-fold stratified cross-validation to preserve class distribution across all folds. For each fold, the dataset is split into training and test subsets, which are processed independently throughout the pipeline. Deep feature extraction is performed using a fine-tuned ResNet-18 network. Fine-tuning is applied only on the training subset of each fold, while the test subset remains completely unseen. The output of the global average pooling layer is used to generate fixed-length feature vectors for the training and testing samples, denoted as $Z_{train}$ and $Z_{test}$, respectively.

For the training features, a preprocessing pipeline is applied sequentially. Feature normalization is first carried out using a StandardScaler fitted exclusively on $Z_{train}$. Dimensionality reduction is then performed using PCA, which is also learned from the training data. To mitigate class imbalance, the Synthetic Minority Over-sampling Technique (SMOTE) is applied after PCA to generate synthetic samples for minority classes. This preprocessing strategy ensures that the classifiers are trained on balanced feature representations. The test features follow a transform-only path. The StandardScaler and PCA models learned from the training data are applied to $Z_{test}$ without re-fitting, and no oversampling is performed on the test set. This design strictly prevents information leakage from the test data into the training process.

Classifier training is conducted using three tree-based models: Decision Tree, Random Forest, and XGBoost. The trained classifiers are then used to generate predictions for the processed test features. Model performance is evaluated using standard classification metrics, including accuracy, precision, recall, F1-score, and confusion matrices. All experimental outputs, including trained models, extracted features, evaluation plots, and execution logs, are saved to support reproducibility and further analysis. In addition, timing measurements are recorded for key pipeline stages, enabling an assessment of computational efficiency. The results are finally visualized through plots and summarized in reports, concluding the evaluation process.

### 3.2. Deep Feature Extraction

Deep feature extraction transforms raw input images into high-level numerical representations that capture visual patterns relevant to classification. Instead of operating directly on pixel-level information, a convolutional neural network is employed to learn hierarchical features that encode semantic characteristics such as edges, textures, shapes, and structural abnormalities.

**Figure 2:**
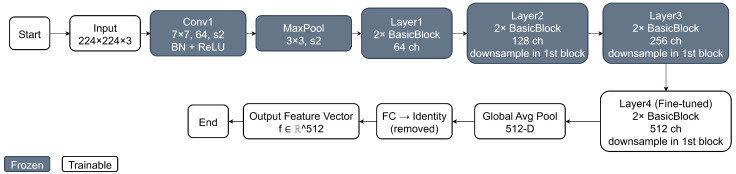
Overall architecture of the deep feature–based model for improving tree-based classification on chest X-ray images.

Figure 2 provides a detailed illustration of the ResNet-18 architecture was used for deep feature extraction in this study. As shown in the figure, an input chest X-ray image of size 224×224×3 is first processed by an initial convolutional layer followed by batch normalization, ReLU activation, and max pooling. The network then consists of four sequential residual stages (Layer1–Layer4), each composed of two basic residual blocks with identity shortcut connections. These residual connections enable efficient gradient propagation and stable feature learning across depth.

In this work, the early convolutional layers and the first three residual stages are kept frozen to preserve general-purpose visual representations learned from ImageNet. Only the final residual stage (Layer4), highlighted as trainable in the figure, is fine-tuned using the training data of each cross-validation fold. This selective fine-tuning strategy allows the deep features to adapt to lung disease patterns in chest X-ray images while reducing the risk of overfitting and maintaining training stability.

Following the final residual block, global average pooling is applied to produce a compact 512-dimensional feature vector. The original fully connected classification layer is removed, and the resulting feature vector is used as the input to the subsequent tree-based classifiers. This design ensures that the extracted features are both semantically rich and well-suited for classical machine learning models, enabling an effective integration of deep representation learning with tree-based classification.

In this work, a ResNet-18 [24] convolutional neural network pre-trained on the ImageNet dataset is used as the feature extractor. Given an input image, the network processes the image through a sequence of convolutional layers, where early layers capture low-level visual patterns. In contrast, deeper layers learn increasingly abstract and task-relevant representations. The extracted deep features provide a fixed-length vector representation for each image, reducing the dimensionality of the input while preserving the most informative visual content. These feature vectors enable classical machine-learning classifiers, such as Random Forest and XGBoost, to operate on semantically meaningful inputs rather than raw pixels.

All chest X-ray images are resized to 224×224 pixels during fine-tuning and feature extraction. Because the original images are grayscale (single channel), the same image is duplicated into three channels to match the input format required by the ImageNet-pretrained ResNet-18 model. After that, the images are normalized using the standard ImageNet mean and standard deviation before being fed into the network. No extra data augmentation (such as rotation, flipping, or color changes) is used. Only resizing and normalization are used. This keeps the preprocessing simple, consistent, and stable while adapting the pretrained model to chest X-ray images.

Let $D={\left\{\left(x_{i},y_{i}\right)\right\}}_{i=1}^{N}$ denote the image dataset, where $x_{i}\in R^{H\times W\times C}$ represents an input image and $y_{i}\in \left\{1,\ldots ,K\right\}$ denotes its corresponding class label. A ResNet-18 convolutional neural network pre-trained on the ImageNet dataset is used as the backbone for deep feature extraction.

Let $f_{\theta }\left(\;\cdot \;\right)$ denote the feature mapping learned by the ResNet-18 network with parameters θ. The final classification layer is removed, and the network is truncated at the global average pooling layer. For an input image $x_{i}$, the extracted deep feature vector is defined as in Equation (1)

(1) $$z_{i}=f_{\theta }\left(x_{i}\right),\;z_{i}\in R^{d},$$

where *d* denotes the dimensionality of the extracted feature representation.

Two feature extraction configurations are considered. In the pretrained configuration, the parameter set θ remains fixed, and the network acts as a deterministic feature extractor. In the fine-tuned configuration, only the parameters of the final residual block, denoted as $\theta_{\mathrm{last}}\subset \theta$, are updated using the training subset of the current cross-validation fold.

Fine-tuning is performed by minimizing the cross-entropy loss over the training set, as defined in Equation (2), while keeping all remaining network parameters frozen.

(2) $$L\left(\theta_{\mathrm{last}}\right)=-\frac{1}{N_{\mathrm{train}}}\sum_{i=1}^{N_{\mathrm{train}}}\log p\left(y_{i}|x_{i};\theta_{\mathrm{last}}\right),$$

**Figure 3:**
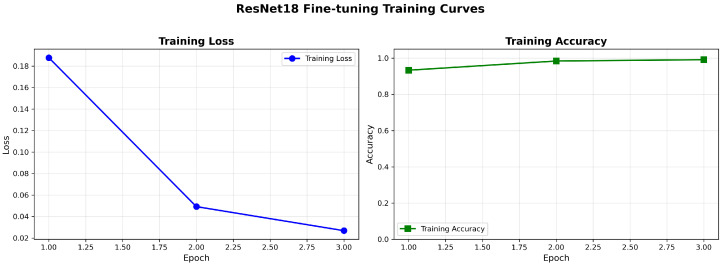
Training loss and accuracy per epoch during fine-tuning of the final residual block for a representative cross-validation fold. The curves show stable convergence within three epochs, with no evident signs of overfitting.

Figure 3 shows the training loss and accuracy per epoch for a representative cross-validation fold. The curves show stable convergence within three epochs, with no clear signs of overfitting. This supports the selected fine-tuning setup (detailed in Section 4.1). Instead of using adaptive early stopping, a fixed training schedule of three epochs was used with a small learning rate of $1\times 10^{-4}$. This allows gentle adaptation of the final residual block while reducing the risk of overfitting in this transfer learning setting.

For each cross-validation fold, feature extraction is conducted independently for the training and test subsets. Let $Z_{\mathrm{train}}\in R^{N_{\mathrm{train}}\times d}$ and $Z_{\mathrm{test}}\in R^{N_{\mathrm{test}}\times d}$ denote the resulting feature matrices for the training and testing sets, respectively. This separation ensures that feature representations used for evaluation are obtained without any influence from the test data during the learning process.

### 3.3. Feature Preprocessing

After deep feature extraction, a preprocessing stage is applied consistently across all experiments to improve feature conditioning and address class imbalance. Let $Z_{\mathrm{train}}\in R^{N_{\mathrm{train}}\times d}$ and $Z_{\mathrm{test}}\in R^{N_{\mathrm{test}}\times d}$ denote the deep feature matrices extracted from the training and test sets, respectively. The training features are first standardized using z-score normalization. For each feature dimension *j*, the normalized feature value is computed as in Equation (3)

(3) $${\tilde{z}}_{ij}=\frac{z_{ij}-\mu_{j}}{\sigma_{j}},$$

where $\mu_{j}$ and $\sigma_{j}$ denote the mean and standard deviation of the *j*-th feature computed from the training data. The same normalization parameters are then applied to the test features.

To reduce feature dimensionality and redundancy, PCA is applied after normalization with a fixed number of components $d^{\prime }=90$ using a randomized singular value decomposition (SVD) solver with a fixed random seed of 42, consistently across all five folds. Given the normalized training feature matrix ${\tilde{Z}}_{\mathrm{train}}$, PCA computes a projection matrix $W\in R^{d\times d^{\prime }}$ that maps the original feature space to a lower-dimensional subspace with $d^{\prime }<d$. The transformed features are obtained as in Equation (4)

(4) $$Z_{\mathrm{train}}^{\prime }={\tilde{Z}}_{\mathrm{train}}W,\;Z_{\mathrm{test}}^{\prime }={\tilde{Z}}_{\mathrm{test}}W,$$

where, for each cross-validation fold, the projection matrix W is learned exclusively from the training data of that fold and then applied unchanged to the corresponding test data. This configuration was selected empirically as a trade-off between retaining most of the discriminative variance in the deep features and keeping the dimensionality manageable for the subsequent tree-based classifiers and was kept fixed across all folds without per-fold hyperparameter tuning.

To mitigate class imbalance, the SMOTE is applied to the reduced training feature set $Z_{\mathrm{train}}^{\prime }$. Within each cross-validation fold, feature normalization and dimensionality reduction are first learned using only the training data. SMOTE is then applied to the transformed training features to generate additional samples for minority classes by interpolating between nearest neighbors in the feature space (with k=5 and a fixed random seed of 42). The resulting balanced training set is denoted as ${\hat{Z}}_{\mathrm{train}}$.

The test data are not subjected to oversampling and are processed solely by applying the normalization and PCA transformations learned from the corresponding training fold. This design prevents information leakage between training and test partitions.

### 3.4. Classification Models

After feature extraction and preprocessing, the resulting feature representations are used to train classical supervised classifiers. Let ${\hat{Z}}_{\mathrm{train}}\in R^{\hat{N}\times d^{\prime }}$ denote the processed training feature matrix obtained after normalization, dimensionality reduction, and class balancing, where $\hat{N}$ represents the number of training samples after oversampling and $d^{\prime }$ denotes the reduced feature dimensionality after PCA. Let $Z_{\mathrm{test}}^{\prime }\in R^{N_{\mathrm{test}}\times d^{\prime }}$ denote the corresponding processed test feature matrix. The associated class labels for the training data are denoted by $y_{\mathrm{train}}\in {\left\{1,\ldots ,K\right\}}^{\hat{N}}$.

#### 3.4.1. Decision Tree Classifier

The Decision Tree classifier learns a hierarchical partitioning of the feature space by recursively selecting feature thresholds that maximize node purity. At each internal node, the optimal split is selected by minimizing an impurity measure such as the Gini index, defined as in Equation (5).

(5) $$\mathrm{Gini}=1-\sum_{k=1}^{K}p_{k}^{2},$$

where $p_{k}$ denotes the proportion of samples belonging to class *k* at a given node. The tree structure induces a piecewise constant decision function that maps an input feature vector z to a predicted class label $\hat{y}\in \left\{1,\ldots ,K\right\}$.

#### 3.4.2. Random Forest Classifier

The Random Forest classifier extends the Decision Tree model by constructing an ensemble of *M* decision trees trained on bootstrapped subsets of the training data. Each tree is trained independently using random feature selection at each split to promote diversity. The prediction for a test sample z is obtained through majority voting over the ensemble as in Equation (6).

(6) $$\hat{y}=\arg\max_{k}\sum_{m=1}^{M}I\left(h_{m}\left(z\right)=k\right),$$

where $h_{m}\left(\;\cdot \;\right)$ denotes the decision function of the *m*-th tree and I(·) is the indicator function. This ensemble strategy improves generalization by reducing variance compared to a single decision tree.

#### 3.4.3. XGBoost Classifier

XGBoost is a gradient-boosted decision tree model that constructs an additive ensemble of weak learners in a sequential manner. At iteration *t*, the model prediction for a sample $z_{i}$ is given, as in Equation (7).

(7) $${\hat{y}}_{i}^{\left(t\right)}=\sum_{j=1}^{t}f_{j}\left(z_{i}\right),$$

where each $f_{j}$ represents a regression tree learned to correct the errors of the previous ensemble.

Training is performed by minimizing a regularized objective function, as in Equation (8).

(8) $$L=\sum_{i}\ell \left(y_{i},{\hat{y}}_{i}\right)+\sum_{j}\Omega \left(f_{j}\right),$$

where ℓ(·) is a differentiable loss function for multiclass classification and Ω(·) is a regularization term that penalizes model complexity. This formulation enables efficient optimization while controlling overfitting.

All classifiers are trained independently for each cross-validation fold using identical feature representations, ensuring a fair and consistent comparison across models.

### 3.5. Datasets

This study combines four datasets originating from different sources into a unified dataset to evaluate the proposed framework with multiclass chest X-ray classification. The primary motivation for integrating multiple datasets is to increase data diversity, reduce dataset-specific bias, and improve the generalizability of learned representations across heterogeneous imaging conditions.

The merged dataset covers five clinical categories: COVID-19, Pneumonia, Tuberculosis, Lung Cancer, and Normal. Four publicly available chest X-ray datasets are utilized as image sources: Dataset 1: COVID-19 Radiography Dataset [25], Dataset 2: Tuberculosis Chest X-ray Dataset [26], Dataset 3: COVID-19, Pneumonia, and Normal Classification Dataset [27], and Dataset 4: Lung Cancer X-ray Dataset [28]. The proposed framework is evaluated on the merged dataset collected from multiple sources. This allows us to evaluate classification performance under different imaging conditions. The number of images in each class of the combined dataset is shown in Table 2.

The chest X-ray images are publicly available and already labeled as one of five classes: COVID-19, Pneumonia, Tuberculosis, Lung Cancer, or Normal. We did not remove any images based on quality, and no extra cleaning was performed. All images were used exactly as provided by the original datasets. They were processed using the same resizing and normalization steps to ensure consistency across all sources.

Class labels in the merged dataset are inherited directly from the original public repositories, with labeling criteria (e.g., clinical diagnosis or radiology report) defined in the cited sources [25,26,27,28]. No relabeling or additional process was performed in this study and images are used with the annotations provided by the dataset curators.

## 4. Results and Discussion

This section reports the performance of the proposed hybrid CNN–tree framework using average results obtained from five-fold cross-validation on the merged dataset. In addition, a strict cross-source evaluation is conducted to assess generalization under domain shift. The evaluation examines the impact of feature representation and preprocessing on the performance of tree-based classifiers in a multiclass chest X-ray classification setting. Three representative models, Decision Tree, Random Forest, and XGBoost, are considered under two configurations: (i) pretrained deep features used directly without additional preprocessing and (ii) fine-tuned deep features combined with feature normalization, dimensionality reduction, and class balancing.

Model performance is reported using accuracy, along with macro-averaged and weighted precision, recall, and F1-score, as well as per-class precision, recall, and F1-scores. For all averaged metrics, the mean and standard deviation across the five folds are provided. Confusion matrices are used to analyze class-wise prediction behavior and common misclassification patterns. In addition, computational efficiency is evaluated by measuring per-sample inference time, providing insight into the practical feasibility of the proposed framework.

### 4.1. Model Configuration and Hyperparameter Settings

To ensure full reproducibility and transparency of the experimental setup, the configuration of the deep feature extractor and the hyperparameters of the tree-based classifiers are explicitly documented in Table 3 and Table 4, respectively.

Table 3 summarizes the fine-tuning strategy for the ResNet-18 model used for deep feature extraction. Rather than fine-tuning the entire network, only the final residual block (layer 4) is updated using the training data of each cross-validation fold, while all preceding layers remain frozen. This selective fine-tuning strategy allows the network to learn chest X-ray features while preserving the general visual patterns learned from large image datasets. Removing the final classification layer and using global average-pooled features yields compact 512-dimensional representations well suited for downstream tree-based learning. The use of a small learning rate together with a limited number of training epochs supports stable optimization and reduces the risk of overfitting.

Table 4 reports the hyperparameter settings used for the Decision Tree, Random Forest, and XGBoost classifiers that were used in this research. All models are configured with common, computationally efficient settings to facilitate fair comparison and practical deployment. The Decision Tree serves as a baseline learner, while Random Forest leverages ensemble learning through bootstrap aggregation to improve robustness and reduce variance. XGBoost employs gradient boosting with shallow trees and controlled learning rates to balance predictive performance and inference efficiency. Importantly, no aggressive hyperparameter tuning is performed to ensure that observed performance gains are primarily due to improved feature representations rather than classifier-specific optimization.

### 4.2. Decision Tree Performance

**Figure 4:**
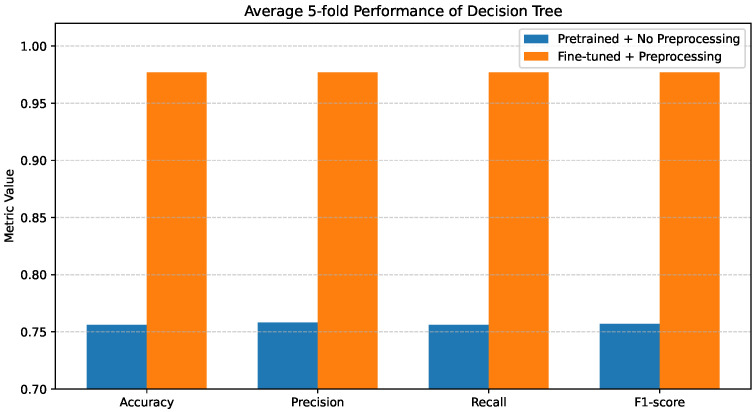
Average 5-fold performance of the Decision Tree classifier under pretrained features without preprocessing and fine-tuned features with preprocessing. Metrics shown are accuracy and weighted precision, recall, and F1-score.

Figure 4 illustrates the average 5-fold performance of the Decision Tree classifier under the two evaluated configurations. When pretrained features are used without preprocessing, the model exhibits relatively weak performance, with Accuracy, Precision, Recall, and F1-score all confined to the range of 0.75–0.76. This outcome reflects limited discriminative capability and a strong sensitivity to noisy or unrefined feature representations.

**Figure 5:**
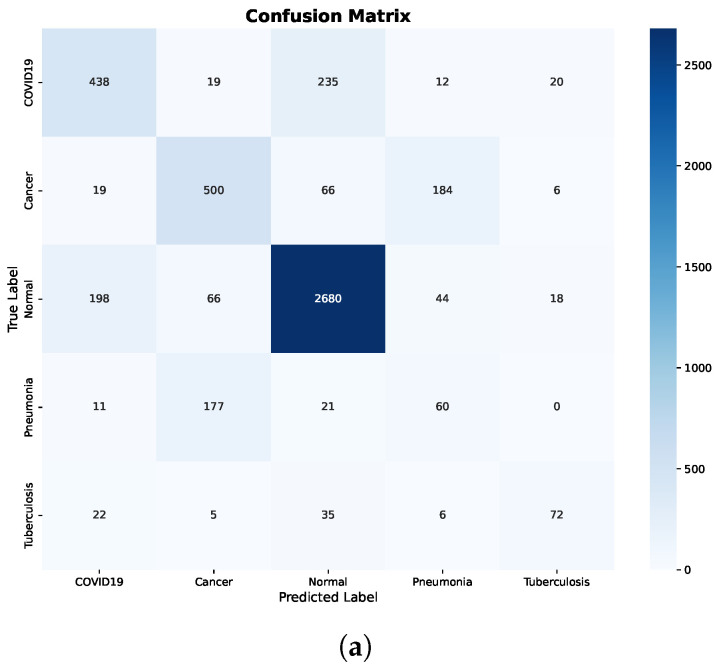

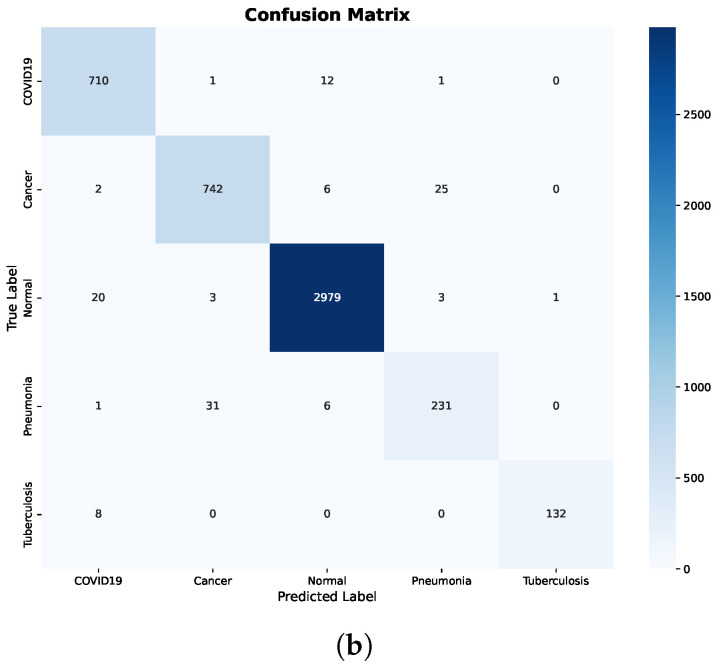
Decision Tree confusion matrices before and after preprocessing. (**a**) Before preprocessing. (**b**) After preprocessing.

Further insight is provided by the confusion matrix shown in Figure 5a. Significant inter-class confusion is observed, particularly between COVID-19 and Normal cases, as well as between Cancer and Pneumonia. Specifically, a substantial number of COVID-19 samples are misclassified as Normal, while Normal samples are frequently misclassified as COVID-19 or Cancer. These error patterns indicate that the Decision Tree struggles to establish reliable decision boundaries when raw, pretrained features are used directly, resulting in reduced recall for clinically critical classes.

In contrast, applying fine-tuning and preprocessing results in substantial and consistent improvements across all evaluation metrics. The confusion matrix after preprocessing, shown in Figure 5b, confirms this improvement, where predictions are predominantly concentrated along the diagonal and off-diagonal misclassifications are markedly reduced.

Importantly, class-level errors are minimized across all disease categories, including COVID-19, Cancer, Pneumonia, and Tuberculosis. The marked reduction in confusion between Normal and pathological classes demonstrates that preprocessing effectively enhances feature separability. Overall, these findings highlight the strong dependence of Decision Trees on feature quality and show that, without appropriate preprocessing, the model tends to underfit and fails to capture meaningful class boundaries.

### 4.3. Random Forest Performance

**Figure 6:**
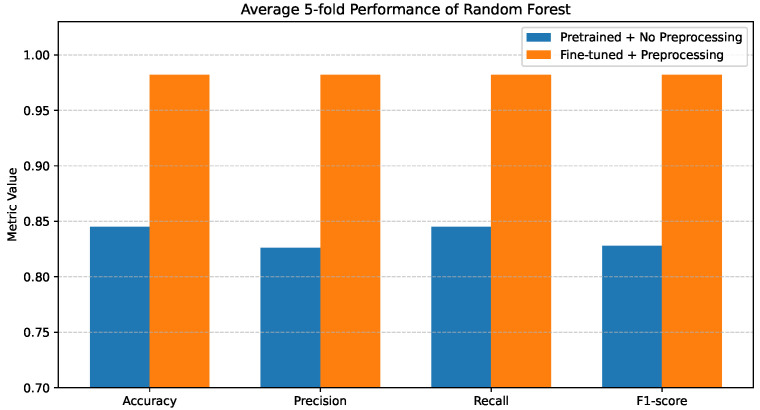
Average 5-fold performance of the Random Forest classifier under pretrained features without preprocessing and fine-tuned features with preprocessing. Metrics shown are accuracy and weighted precision, recall, and F1-score.

The Random Forest results are presented in Figure 6. When pretrained features are used without preprocessing, the model demonstrates improved robustness compared to the single Decision Tree, achieving Accuracy and Recall values of 0.84–0.85, while Precision and F1-score remain slightly lower at 0.82–0.83.

This improvement is attributed to the ensemble learning mechanism of Random Forest, which reduces variance and improves generalization.

**Figure 7:**
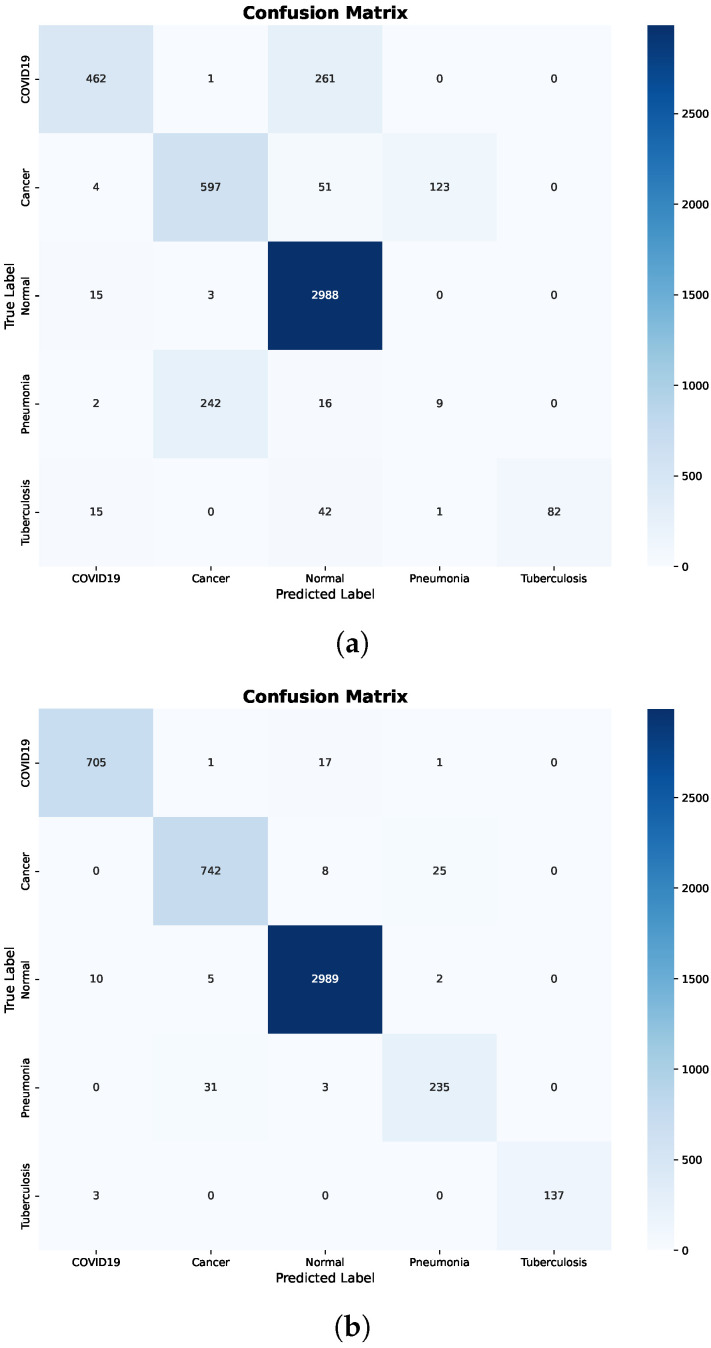
Random Forest confusion matrices before and after preprocessing. (**a**) Before preprocessing. (**b**) After preprocessing.

Additional insight is provided by the confusion matrix shown in Figure 7a. While the overall classification behavior is more stable than that of the Decision Tree, noticeable inter-class confusion remains. In particular, confusion persists between COVID-19 and Normal cases, as well as between Cancer and Pneumonia. These misclassifications suggest that, although ensemble learning alleviates overfitting, raw pretrained features still limit the model’s ability to fully separate clinically similar disease classes.

Random Forest achieves consistently high performance across all evaluation metrics when using fine-tuned and preprocessed features. The corresponding confusion matrix in Figure 7b confirms this improvement, where predictions are strongly aligned along the diagonal and inter-class errors are substantially reduced.

### 4.4. XGBoost Performance

**Figure 8:**
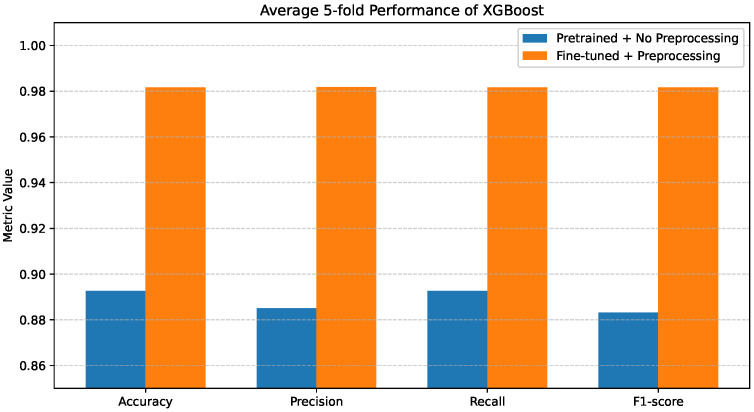
Average 5-fold performance of the XGBoost classifier under pretrained features without preprocessing and fine-tuned features with preprocessing. Metrics shown are accuracy and weighted precision, recall, and F1-score.

Figure 8 reports the average 5-fold performance of the XGBoost classifier under the two evaluated configurations. When pretrained features are used without preprocessing, XGBoost outperforms both Decision Tree and Random Forest in the same setting, achieving Accuracy, Precision, Recall, and F1-score values of 0.88–0.89.

**Figure 9:**
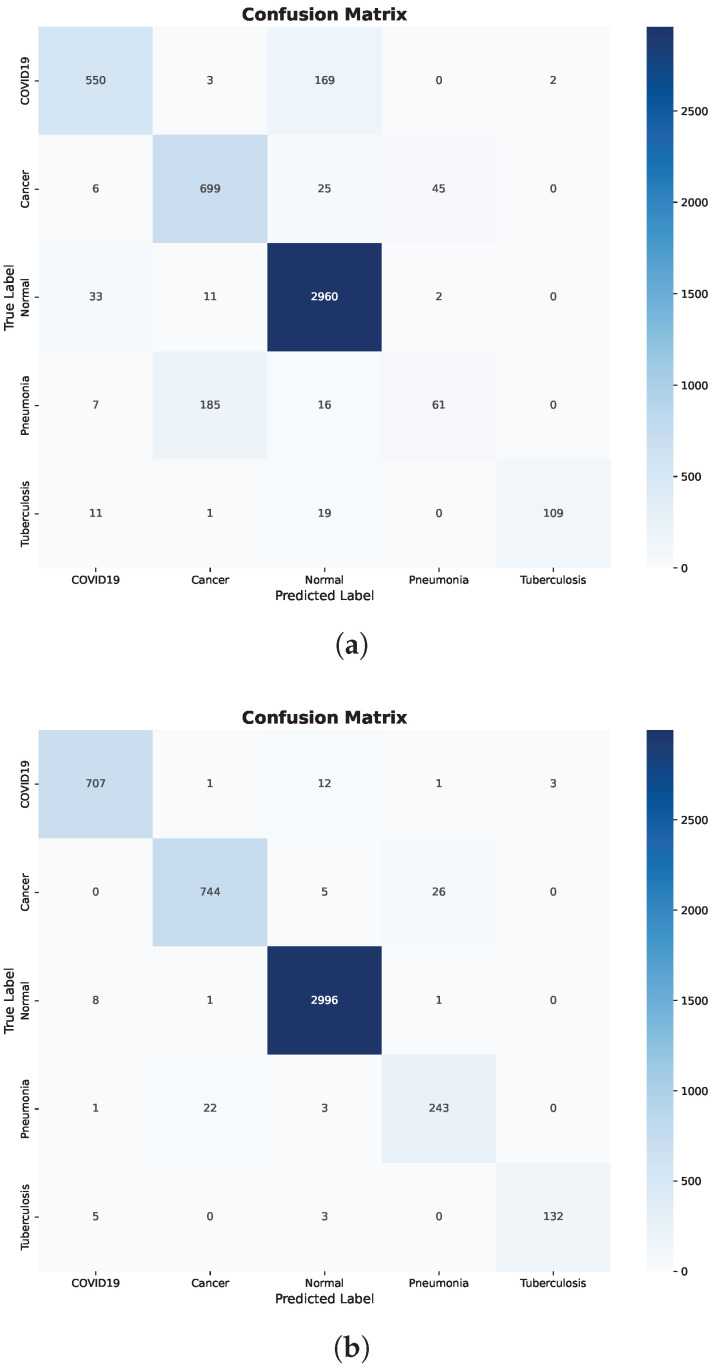
XGBoost confusion matrices before and after preprocessing. (**a**) Without preprocessing. (**b**) With preprocessing.

Figure 9a shows further insights using the confusion matrix. Although XGBoost exhibits lower inter-class confusion than the other classifiers without preprocessing, notable misclassifications persist, particularly for COVID-19 and Pneumonia cases. These patterns indicate that, despite its robustness, the lack of preprocessing still limits the model’s ability to separate clinically overlapping disease categories fully.

When fine-tuning and preprocessing are applied, XGBoost attains near-optimal performance across all evaluation metrics. The corresponding confusion matrix in Figure 9b confirms this improvement, where predictions are highly concentrated along the diagonal and inter-class misclassifications are minimal.

Table 5 summarizes the average classification performance obtained using five-fold cross-validation for the fine-tuned and preprocessed configuration. Reported values correspond to the mean and standard deviation across folds. Performance is evaluated using accuracy, along with macro-averaged and weighted precision, recall, and F1-score. While macro-averaged metrics assign equal importance to each class, weighted metrics account for the underlying class distribution. Per-class precision, recall, and F1-scores for individual folds are provided in the per-fold result files.

Across all three tree-based classifiers, the results in Table 5 indicate that accuracy, macro-averaged, and weighted metrics consistently approach between 0.958 and 0.982 with small standard deviations across the five folds. The consistently high macro-averaged scores suggest that performance gains extend beyond the predominant Normal class to minority disease categories. Together with the per-class metrics and ROC-AUC values obtained for each fold, these findings indicate stable performance across cross-validation splits rather than dependence on a single favorable data partition.

### 4.5. Per-Class Performance, PR-AUC, and Calibration Analysis

In addition to the per-class precision, recall, and F1-score results, we also include evaluation using precision–recall curves and probability calibration, as listed in Table 6. These metrics help us better understand how the classifiers perform when the classes are imbalanced. They also show how reliable the predicted confidence scores are, going beyond simple accuracy.

Precision–Recall Area Under the Curve (PR-AUC) is calculated for each class across the five cross-validation folds. Random Forest and XGBoost show consistently high macro PR-AUC values with very small variation between folds. This means they perform stably even when the classes are imbalanced. The Decision Tree has slightly lower macro PR-AUC values, which suggests that ensemble methods separate similar disease classes more effectively. For individual classes, the Normal class achieves almost perfect PR-AUC. Pneumonia is more difficult to classify because it looks visually similar to other lung diseases. The small variation between folds shows that the results are consistent and not dependent on one specific data split.

**Figure 10:**
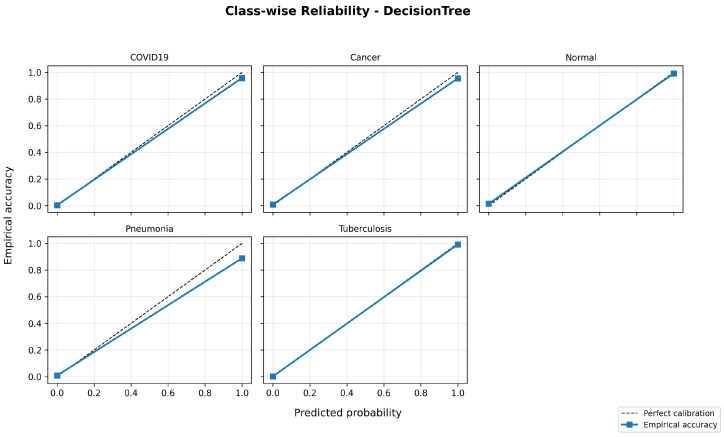
Class-wise reliability diagram for the Decision Tree classifier (representative fold).

**Figure 11:**
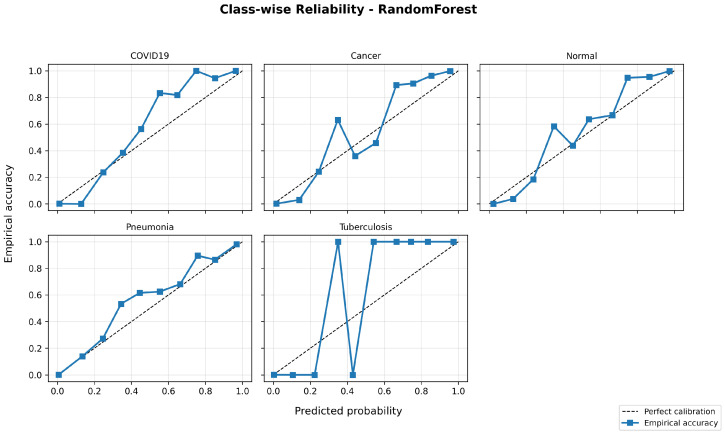
Class-wise reliability diagram for the Random Forest classifier (representative fold).

**Figure 12:**
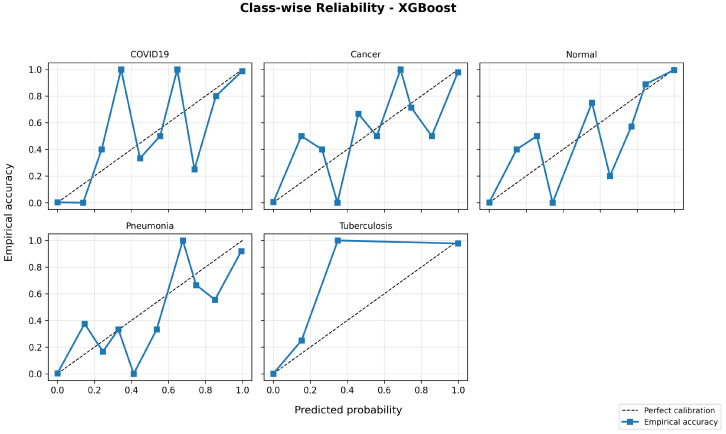
Class-wise reliability diagram for the XGBoost classifier (representative fold).

Calibration quality is further evaluated using reliability diagrams and multi-class Brier scores to examine the alignment between predicted probabilities and empirical accuracy. Figure 10, Figure 11 and Figure 12 show representative calibration curves for each classifier. Random Forest and XGBoost demonstrate improved probability calibration with lower Brier scores compared to the Decision Tree, indicating more reliable confidence estimates. Although some probability bins exhibit variability due to limited sample counts, the overall curves remain close to the ideal diagonal, suggesting that predicted probabilities retain meaningful interpretability.

The inclusion of PR-AUC, reliability diagrams, and calibration metrics provides a more comprehensive assessment of model performance beyond overall accuracy and supports the stability of the reported results across folds.

### 4.6. Qualitative Analysis of CNN Feature Activation (Grad-CAM)

Grad-CAM visualizations are used to provide qualitative insight into the image regions emphasized by the ResNet-18 backbone during deep feature extraction. In the proposed hybrid framework, the CNN serves exclusively as a feature extractor, while the final classification is performed by tree-based models operating on the extracted representations. As a result, the Grad-CAM maps illustrate the focus of the CNN feature extractor rather than the decision mechanism of the downstream classifier.

**Figure 13:**
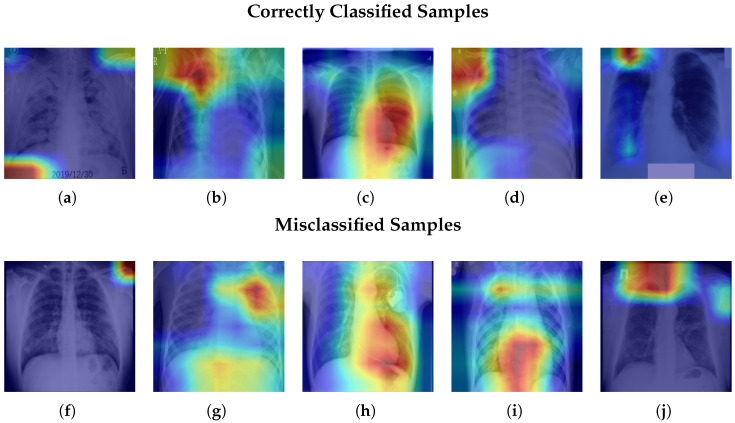
Grad-CAM heatmaps from the ResNet-18 layer4 feature extractor for representative test samples. The top row shows correctly classified cases, while the bottom row shows misclassified cases across all five classes. The highlighted regions indicate areas contributing most strongly to CNN feature activation. (**a**) COVID-19. (**b**) Cancer. (**c**) Normal. (**d**) Pneumonia. (**e**) Tuberculosis. (**f**) COVID-19. (**g**) Cancer. (**h**) Normal. (**i**) Pneumonia. (**j**) Tuberculosis.

Figure 13 presents Grad-CAM heatmaps for representative correct and misclassified test samples from each class. The visualizations are generated using activations and gradients from the layer4 block of the fine-tuned ResNet-18 model and highlight image regions that most strongly influence the learned feature representations.

For correctly classified images, the activation maps usually highlight important areas in the chest, especially the lung regions. Sometimes they also include nearby areas. This shows that the CNN focuses on disease-related patterns in the lungs, not on irrelevant background parts of the image. For misclassified images, the activation maps are more spread out and less focused. The model’s attention is often scattered across areas that are not clearly different. This happens because some lung diseases look very similar. For example, COVID-19 and pneumonia can appear alike, which explains why the model sometimes confuses them.

### 4.7. Feature Importance Analysis of Classifiers

In addition to the Grad-CAM analysis of the CNN feature extractor, we analyze feature importance from the tree-based classifiers. This helps us understand how the extracted deep features affect the final predictions. Because the classifiers use PCA-transformed features, the importance scores refer to principal components, not to individual image pixels.

**Figure 14:**
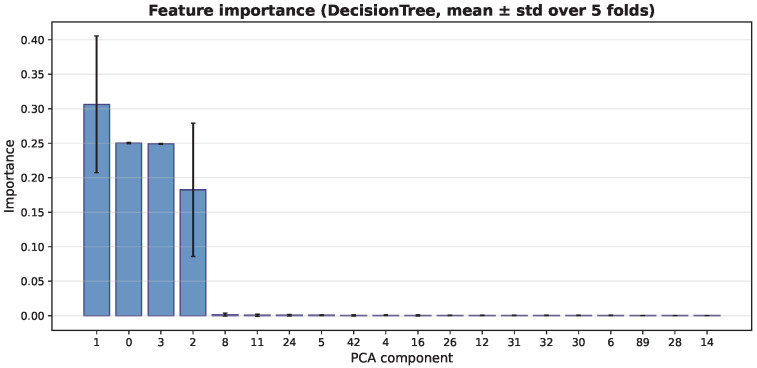
Feature importance of PCA components for the Decision Tree classifier (mean ± std across five folds).

**Figure 15:**
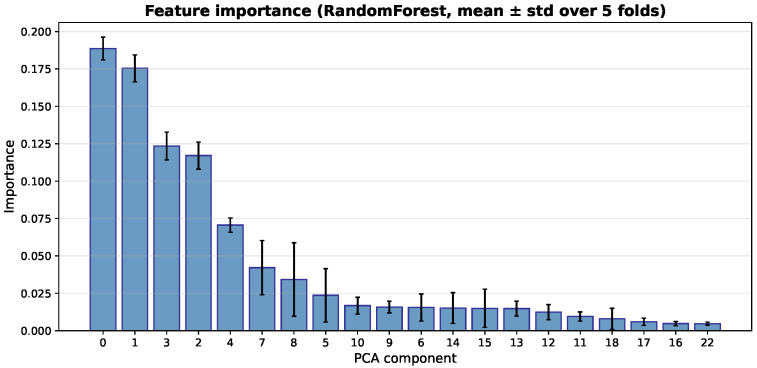
Feature importance of PCA components for the Random Forest classifier (mean ± std across five folds).

**Figure 16:**
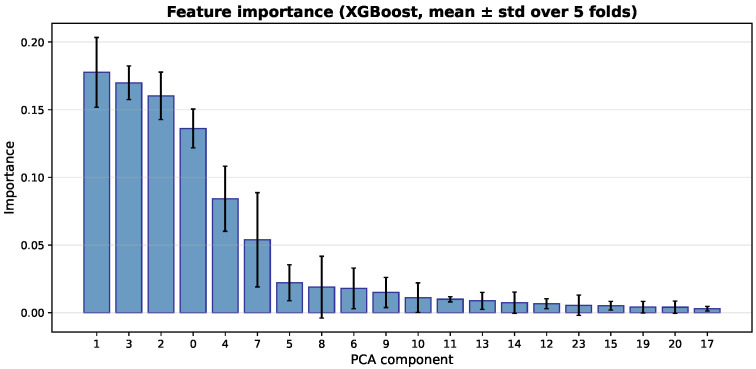
Feature importance of PCA components for the XGBoost classifier (mean ± std across five folds).

Figure 14, Figure 15 and Figure 16 show the average importance of the PCA components across the five cross-validation folds for the three classifiers. The Decision Tree focuses mainly on a few early components. PCA component 1 has the highest importance (average 0.306), followed by components 0 and 3. The other components have very little impact. In contrast, the ensemble models (Random Forest and XGBoost) spread the importance across more components. For Random Forest, component 0 has the highest average importance (0.189), and components 1, 3, and 2 also contribute significantly. This means that several leading components work together to make predictions. XGBoost shows a similar but smoother pattern. Component 1 has the highest importance (0.178), followed by components 3, 2, and 0. This indicates a more balanced use of multiple important components. Overall, Random Forest and XGBoost rely on a wider set of features, while the Decision Tree depends mainly on a small number of components.

Although PCA components are not directly interpretable in spatial terms, combining Grad-CAM visualizations with feature-importance analysis provides complementary interpretability at both the image and representation levels.

### 4.8. Cross-Source Generalization Analysis

To further evaluate robustness under domain shift, a cross-source experiment was conducted in which Dataset 3 [27] was used exclusively for training and Dataset 1 [25] was used exclusively for testing. The two datasets share identical class labels, namely COVID-19, Pneumonia, and Normal. No images from Dataset 1 were used during CNN fine-tuning, feature normalization, PCA fitting, or classifier training. The test set was processed using transform-only operations, applying the StandardScaler and PCA models learned from Dataset 3 without re-fitting, and no oversampling was performed on the test data. This setup enforces strict source separation and provides a realistic assessment of cross-domain generalization.

**Figure 17:**
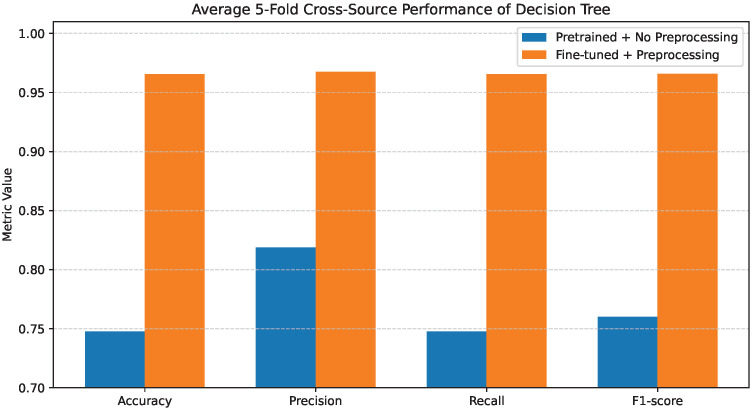
Average 5-fold cross-source performance of Decision Tree.

**Figure 18:**
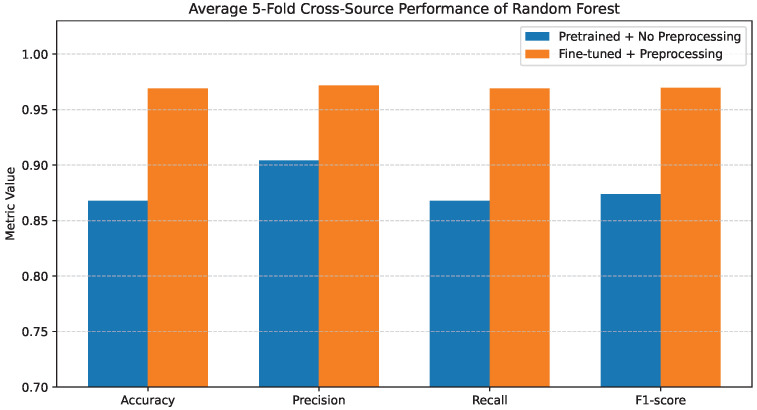
Average 5-fold cross-source performance of Random Forest.

**Figure 19:**
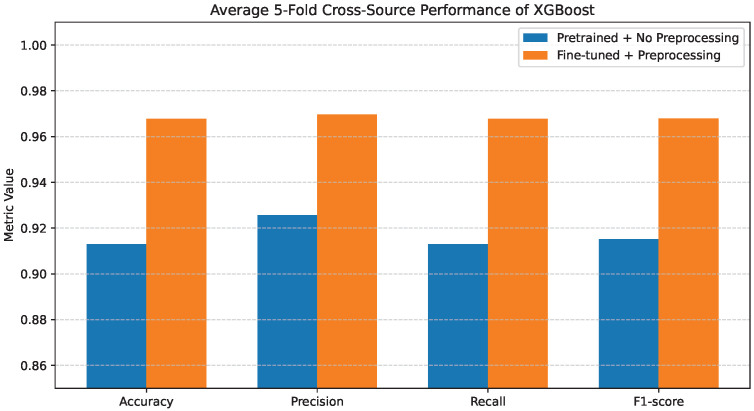
Average 5-fold cross-source performance of XGBoost.

Figure 17, Figure 18 and Figure 19 present the average 5-fold cross-source performance of the Decision Tree, Random Forest, and XGBoost classifiers, respectively. As shown in Figure 17, the Decision Tree classifier experiences a marked reduction in performance under the pretrained configuration without preprocessing. Accuracy and weighted F1-score remain in a lower range, indicating limited robustness when raw pretrained features are directly transferred across sources. When fine-tuning and preprocessing are applied, performance improves consistently across accuracy, weighted precision, recall, and F1-score. The improvement demonstrates that domain-adapted feature representations significantly enhance separability even in the presence of cross-source variability.

Figure 18 shows that Random Forest exhibits greater robustness than a single Decision Tree under cross-source conditions. Even without preprocessing, ensemble averaging reduces variance and mitigates severe performance degradation. However, a clear performance gap remains between the pretrained and fine-tuned configurations. After fine-tuning and preprocessing, all reported metrics increase substantially, indicating that ensemble learning benefits strongly from improved feature conditioning and reduced redundancy. The cross-source results confirm that while ensemble methods enhance stability, feature quality remains the primary determinant of generalization performance.

As illustrated in Figure 19, XGBoost achieves the highest cross-source performance among the evaluated tree-based classifiers. Under the pretrained configuration, it outperforms Decision Tree and Random Forest. This suggests that XGBoost exhibits stronger tolerance to feature distribution shifts. Nevertheless, fine-tuning combined with preprocessing produces further consistent gains across all metrics. The performance differences between classifiers become considerably smaller after applying fine-tuning and preprocessing. This suggests that feature representation quality has a greater impact on cross-source generalization than classifier complexity.

### 4.9. Computational Efficiency

Before presenting the computational efficiency results, it is important to clarify that, in this study, a sample corresponds to a single chest X-ray image resized to 224×224 pixels. Each image is processed independently through the feature extraction, preprocessing, and classification stages when measuring per-sample inference time.

Table 7 summarizes the average end-to-end per-sample inference time of the proposed hybrid CNN–tree framework, reported in milliseconds under the fine-tuned and preprocessed configuration. In this setting, a ResNet-18 backbone is used only for deep feature extraction, while lightweight tree-based models perform classification. The results indicate that deep feature extraction dominates the overall inference cost, accounting for approximately 3.5–3.9 ms per image. This behavior is expected, as convolutional feature extraction is computationally more demanding than the subsequent preprocessing and tree-based prediction stages.

The preprocessing stage, which includes StandardScaler and PCA transformations, introduces negligible overhead, contributing less than 0.06 ms per image across all classifiers. This confirms that normalization and dimensionality reduction can be integrated into the pipeline without adversely affecting inference efficiency.

Classifier prediction time accounts for only a very small fraction of the total inference cost. The Decision Tree exhibits the lowest prediction latency, followed by XGBoost, while Random Forest incurs a slightly higher inference cost due to its ensemble-based structure. Nevertheless, these differences are marginal compared to the cost of deep feature extraction.

Overall, the end-to-end per-sample inference time of the hybrid CNN–tree pipeline remains within a narrow range across all evaluated models, ranging from approximately 3.56 ms to 3.97 ms. Random Forest achieves the lowest total inference time, followed closely by XGBoost and Decision Tree. These results demonstrate that the proposed framework maintains high computational efficiency while delivering strong classification performance, supporting its suitability for real-time or near-real-time clinical decision-support systems.

To further analyze the contribution of the CNN backbone, we examine the per-sample inference cost and feature quality of different convolutional networks when used for direct end-to-end CNN classification. In terms of computational efficiency, the total per-sample inference time remains low and consistent across all evaluated backbones, ranging from 17.33 ms to 18.41 ms per image, as reported in Table 8. This narrow range indicates that the backbone choice has only a marginal impact on inference latency when CNNs are used as end-to-end classifiers.

From a performance perspective, EfficientNet-B0 achieves the highest classification effectiveness, with an accuracy of 0.9921 and a Macro-F1 score of 0.9883, followed closely by ResNet-18 with a Macro-F1 score of 0.9845. Lightweight architectures such as ShuffleNetV2 and MobileNetV3 exhibit lower performance, while SqueezeNet1_1 shows a noticeable degradation in feature quality, reflected by its reduced Macro-F1 score of 0.8943. These results indicate that overly compact models sacrifice discriminative feature capacity despite offering slightly lower inference times.

Although SqueezeNet1_1 and ShuffleNetV2 achieve the lowest CNN inference latency, their reduced feature quality leads to inferior classification performance compared to more expressive backbones. EfficientNet-B0 offers the best trade-off between feature quality and computational cost among CNN-only models, delivering the strongest predictive performance with only a marginal increase in inference time.

However, compared with the proposed hybrid CNN–tree framework, end-to-end CNN classifiers are substantially more computationally demanding. While CNN-only models require approximately 17–18 ms per image, the hybrid pipeline achieves end-to-end inference times of only 3.56–3.97 ms by replacing the CNN classification head with lightweight tree-based predictors. This represents an approximately 5-fold reduction in inference cost while preserving comparable predictive performance, highlighting the efficiency advantage of the proposed architecture for real-time, resource-constrained clinical deployment.

EfficientNet-B0 employs a compound scaling strategy that jointly scales network depth, width, and input resolution [29], achieving slightly higher accuracy and Macro-F1 scores. However, the performance difference compared to ResNet-18 is marginal. While EfficientNet-B0 can be fine-tuned, its coupled architectural design provides less explicit modular block-wise control than residual architectures. In contrast, ResNet-18 is constructed from clearly defined residual blocks connected through identity shortcut connections, which enable learning residual functions and have been shown to ease optimization and improve training stability [24]. This residual structure enables precise, controlled fine-tuning of the final network block, making ResNet-18 more suitable for reproducible deep feature extraction and consistent feature adaptation in tree-based classification pipelines.

The experimental results demonstrate that the quality of deep feature representations is the primary determinant of performance in tree-based chest X-ray classification. Across all evaluated classifiers, fine-tuning combined with feature preprocessing leads to a substantial and consistent improvement in accuracy, precision, recall, and F1-score, while significantly reducing inter-class confusion. In contrast, using raw pretrained features results in unstable decision boundaries and degraded performance, even for ensemble models.

Once feature representations are optimized, the performance gap between Decision Tree, Random Forest, and XGBoost becomes negligible, indicating that classifier complexity plays a secondary role compared to feature quality. Ensemble methods improve robustness but cannot compensate for poorly conditioned features. Selective fine-tuning of the CNN backbone yields significant accuracy gains with minimal computational overhead, preserving inference efficiency.

Overall, the results confirm that the proposed CNN-tree framework effectively balances classification performance, computational efficiency, and reproducibility, making it suitable for multiclass chest X-ray analysis and for potential deployment in real-time or near-real-time clinical decision-support systems. At the same time, the reported high cross-validation performance should be interpreted as an upper-bound estimate within the merged multi-dataset benchmark. Generalization to new institutions may vary due to differences in acquisition protocols, imaging conditions, and the methodological limitations discussed in Section 3.5 and Section 3.

### 4.10. Comparison with Existing Methods

Table 9 compares the proposed framework with recent studies on automated lung disease analysis using chest imaging. The comparison focuses on classification performance and computational requirements, both of which are critical for practical clinical deployment.

Several existing studies emphasize training time as a key comparison factor, such as the YOLOv8-based framework by A Hasib et al. [16] and the transfer learning study by Mirugwe et al. [21]. However, training time is typically an offline process and does not directly affect daily clinical use. In real-world screening systems, inference time and prediction reliability are more important, especially when models are deployed in high-volume or resource-limited settings.

The proposed framework reaches an F1 score of 0.982 with a low inference time of 3.56–3.97 ms per image. Unlike end-to-end deep learning models, inference does not require heavy resources, as classification is performed using lightweight tree-based models.

Compared to ETDHDNet proposed by Shati et al. [17], which reports an inference time of 0.7–2 ms, the proposed method achieves a higher F1-score (0.982 versus 0.973) while supporting multiclass classification across five lung disease categories. This shows that the proposed framework improves diagnostic coverage and accuracy with only a small increase in inference time.

Overall, the results indicate that the proposed hybrid CNN–tree framework provides a good balance between accuracy and computational efficiency, making it suitable for practical chest X-ray screening applications.

### 4.11. Dataset Limitations

Although the merged multi-source dataset increases diversity and enables evaluation under heterogeneous imaging conditions, several limitations should be acknowledged. First, the original datasets do not provide unified patient identifiers; therefore, the evaluation is performed at the image level rather than with patient-wise grouping. As a result, strict patient-level separation across all sources cannot be guaranteed. Second, no explicit cross-dataset deduplication or near-duplicate filtering was conducted, and a residual risk of overlap between sources may remain.

Third, the datasets originate from different institutions and acquisition environments, and metadata regarding projection views, posteroanterior (PA) and anteroposterior (AP), is not consistently available. Images were included as released, without filtering by view. Variations in scanner settings, image contrast, borders, and other non-diagnostic elements may introduce dataset-specific characteristics. These limitations arise from the structure and metadata constraints of the original public repositories and reflect the conditions under which the data were released. While such variability reflects real-world clinical scenarios, it may also allow models to partially rely on acquisition-related cues rather than purely pathological patterns.

## 5. Conclusions

This study presents an effective hybrid framework for improving the performance of tree-based classifiers in multiclass chest X-ray image classification. By integrating deep feature extraction with classical tree-based learning, the proposed approach overcomes the inherent limitations of decision-tree models when applied directly to high-dimensional medical images. A fine-tuned ResNet-18 network was employed to extract compact and discriminative feature representations, which were subsequently enhanced through normalization, dimensionality reduction, and class balancing before classification. Extensive experiments conducted using stratified five-fold cross-validation on a heterogeneous, multi-source chest X-ray dataset demonstrated that fine-tuned deep features combined with appropriate preprocessing show consistent performance improvements across Decision Tree, Random Forest, and XGBoost classifiers. Under this configuration, all evaluated tree-based models achieved weighted F1-scores between 0.977 and 0.982 across five-fold cross-validation, with a reduction in inter-class confusion, particularly among clinically similar disease categories such as COVID-19, pneumonia, and normal cases. In contrast, models trained on raw pretrained features exhibited noticeably lower performance and unstable decision boundaries. These findings confirm that feature representation quality plays a more decisive role than classifier complexity in achieving reliable tree-based performance for medical imaging tasks. Computational efficiency analysis further confirmed that the proposed framework maintains low inference latency, with end-to-end per-sample inference times remaining below 4 ms. The dominant computational cost arises from the deep feature extraction stage, while preprocessing and classifier inference introduce negligible overhead. The efficiency of tree-based models supports the suitability of the framework for real-time or near-real-time clinical decision-support systems and large-scale screening scenarios. Future work will extend this framework to support disease severity grading across merged lung X-ray datasets. Rather than restricting predictions to categorical diagnoses, the learned deep feature representations will be leveraged to model disease severity levels and progression patterns, enabling longitudinal monitoring and more comprehensive clinical assessment.

## Figures and Tables

**Table 1:** Summary of related works.

Authors (Ref)	Year	Methods	Datasets Used	Computational Cost	Accuracy	Limitation
Lu et al. [15]	2025	Vision Transformer, Patch Reduction Block & Randomized Classifier	COVID-19 & Pneumonia and COVID-19 Radiography	Used 60% of the tokens that reduced the consumption of training time and computing resources	98.4–98.5%	Performance may be limited when applied directly to high-dimensional images without token reduction.
Shati et al. [17]	2025	ETDHDNet (DenseNet121 + Extended Texture Descriptor Histogram)	TBX11K, Shenzhen, Qatar Univ. TB Database	0.7–2 ms inference time	88.05–99.29%	Accuracy may drop on more complex/diverse datasets.
A Hasib et al. [16]	2025	YOLOv8 framework with FILM & PCA synthetic augmentation	COVID-19 & Pneumonia dataset	Total training: 2820 s (YOLOv8) vs 3753 s (InceptionV3)	96–97%	Synthetic data may lack sufficient diversity to replicate rare pathologies.
Randieri et al. [18]	2025	Lightweight CNN (19 layers) with CLAHE & Data Augmentation	the COVID-19, Pneumonia and Normal Chest X-ray PA and COVID-19 Radiography Database	Inference time: $9.92\times 10^{-4}$ s	97.48%	Relies on balanced datasets
Alotaibi et al. [19]	2025	Modified Pre-trained ResNet-50	Local dataset, COVID-19-radiography-database and Chest X-Ray Images (Pneumonia)	Not reported	95.28%	Direct application to high-dimensional images without preprocessing leads to saturation at ∼30 epochs.
Wajgi et al. [20]	2024	VGG19 Transfer Learning + Hyperparameter Tuning (Tanh + SGD)	TB Chest X-ray Database	Not reported	98.11%	Accuracy drops significantly to 92.12% when preprocessing is skipped.
Mirugwe et al. [21]	2025	Comparison of 6 architectures (VGG, ResNet, Inception-ResNet)	Tuberculosis (TB) Chest X-ray Database	training the ResNet152 with data augmentation took 356.6 min	99.4% (VGG16)	Deeper models like ResNet152 increase resource demand without proportional accuracy gains.
Rahman et al. [14]	2024	TB-CXRNet (CheXNet encoder + Self-MLP classifier)	QU-MLG-TB	Not reported	93.32%	Direct classification on high-dimensional raw images is prone to subjectivity and expert dependence.
Srinivas et al. [22]	2024	Hybrid IV3–VGG (Inception V3 + VGG16)	COVID-19 Radiography Database	Not reported	98%	Overfitting and false predictions may occur for poor-quality CXRs without the hybrid approach.
El Houby [23]	2024	Transfer Learning (VGG19 & EfficientNetB0) with HE/CLAHE	COVID-19 Radiography Database	Not reported	95%	Performance is limited when using segmented lungs due to loss of contextual information.

**Table 2:** Per-class distribution of chest X-ray images in the combined dataset.

Class	Number of Images
COVID-19	3616
Pneumonia	1345
Tuberculosis	700
Lung Cancer	3875
Normal	15,033
**Total**	24,569

**Table 3:** ResNet-18 fine-tuning configuration used for deep feature extraction.

Parameter	Setting
Architecture	ResNet-18 (ImageNet pretrained)
Input Image Size	224×224 RGB
Frozen Layers	All convolutional layers except the final residual block
Fine-tuned Layers	Residual block layer4 only
Fully Connected Layer	Removed and replaced with identity mapping
Feature Dimension	512 (global average pooled features)
Optimizer	Adam
Learning Rate	$1\times 10^{-4}$
Loss Function	Cross-Entropy Loss
Training Epochs	3
Batch Size	32

**Table 4:** Hyperparameters of tree-based classifiers used for classification.

Classifier	Hyperparameters
Decision Tree	Split criterion: Gini; maximum depth: default (unrestricted); random state: 42.
Random Forest	Number of trees: 100; split criterion: Gini; bootstrap sampling: enabled; feature selection: default (auto); parallel jobs: all available cores; random state: 42.
XGBoost	Number of trees: 100; learning rate: 0.1; maximum tree depth: 4; subsample ratio: 0.8; column subsample ratio: 0.8; objective function: multi-class probability estimation; evaluation metric: multi-class log loss; tree construction method: histogram-based; random state: 42.

**Table 5:** Average classification metrics across five-fold cross-validation for the fine-tuned feature representation with preprocessing. Values are reported as mean ± standard deviation.

Classifier	Accuracy	Macro Precision	Macro Recall	Macro F1-Score	Weighted Precision	Weighted Recall	Weighted F1-Score
Decision Tree	0.977±0.001	0.960±0.005	0.958±0.005	0.959±0.005	0.977±0.001	0.977±0.001	0.977±0.001
Random Forest	0.982±0.002	0.969±0.005	0.961±0.004	0.965±0.004	0.982±0.002	0.982±0.002	0.982±0.002
XGBoost	0.982±0.001	0.966±0.004	0.962±0.004	0.964±0.004	0.982±0.001	0.982±0.001	0.982±0.001

**Table 6:** Additional evaluation based on macro PR-AUC and calibration performance across five-fold cross-validation. Values are reported as mean ± standard deviation.

Classifier	Macro PR-AUC	Brier Score
Decision Tree	0.920±0.006	0.045±0.002
Random Forest	0.988±0.002	0.033±0.002
XGBoost	0.987±0.002	0.031±0.002

**Table 7:** End-to-end per-sample inference time of the proposed hybrid CNN–tree pipeline, where ResNet-18 is used for feature extraction and tree-based models perform final classification.

Stage	Decision Tree (ms)	Random Forest (ms)	XGBoost (ms)
ResNet-18 Feature Extraction (without FC head)	3.9188	3.5022	3.8240
Preprocessing (StandardScaler + PCA)	0.0484	0.0511	0.0410
Tree-Based Inference	0.00008	0.00520	0.00073
Total Inference Time	3.97	3.56	3.87

**Table 8:** Per-sample end-to-end inference time and feature quality of different CNN backbone networks used for direct CNN-based classification (5-fold stratified CV).

Backbone	Accuracy	Macro-F1	Total CNN Inference Time (ms)
ResNet-18	0.9908	0.9845	18.41
MobileNetV3	0.9776	0.9622	18.20
EfficientNet-B0	0.9921	0.9883	17.88
ShuffleNetV2 (×1.0)	0.9785	0.9607	17.49
SqueezeNet1_1	0.9407	0.8943	17.33

**Table 9:** Comparison with existing research in automated lung disease imaging.

Authors	Proposed Method	F1-Score	Computation
Lu et al. [15]	CTBViT: Vision Transformer with Patch Reduction Block for tuberculosis detection using chest CT and X-ray images	0.978	Used 60% of the tokens that reduced the consumption of training time and computing resources
A Hasib et al. [16]	YOLOv8-based framework with synthetic image augmentation for COVID-19 and pneumonia detection from chest X-ray images	0.970	Total training: 2820 s (YOLOv8) vs 3753 s (InceptionV3)
Shati et al. [17]	ETDHDNet: DenseNet-based model integrating multi-scale texture descriptors for tuberculosis prediction from chest X-ray images	0.973	0.7–2 ms inference time
Mirugwe et al. [21]	Comparative transfer learning framework using CNN architectures (VGG16, ResNet, Inception-ResNet) for tuberculosis detection from chest X-ray images	0.983	Training the ResNet152 with data augmentation took 356.6 min
**This work**	Fine-tuned ResNet-18 deep feature extraction followed by tree-based classifiers for multiclass chest X-ray classification	**0.977–0.982**	3.56–3.97 ms inference time

## Data Availability

The datasets used in this study are publicly available and can be accessed through the original repositories cited in the manuscript (COVID-19 Radiography, Tuberculosis Chest X-ray, COVID-19 Pneumonia Normal Classification, and Lung Cancer X-ray datasets). Access links and source details are provided in the reference list.
